# 急性髓系白血病微小残留病检测与临床解读中国专家共识（2021年版）

**DOI:** 10.3760/cma.j.issn.0253-2727.2021.11.002

**Published:** 2021-11

**Authors:** 中华医学会血液学分会实验诊断学组

急性髓系白血病（AML）是一种来源于造血干细胞的血液系统恶性疾病，以髓系来源的异常分化原始细胞克隆性扩增为特征，年龄<60岁的患者长期生存率为35％～45％，年龄≥60岁的患者长期生存率仅为10％～15％[1]–[3]。白血病复发是提高AML疗效的主要瓶颈。国内外的研究表明微小残留病（MRD）检测不仅可用于疗效评估、复发预警，还可用于指导治疗方法的选择以及抢先干预[4]–[15]。因此，MRD检测已成为降低白血病复发、提高疗效的关键环节之一。AML患者的MRD检测方法包括多参数流式细胞术（MFC）、实时定量聚合酶链反应（RQ-PCR）技术以及数字PCR（dd-PCR）和二代测序技术（NGS）等，其中dd-PCR和NGS尚处于临床研究阶段，未被常规用于MRD评估[10],[16]–[18]。目前，我国尚缺乏针对AML患者MRD检测以及如何将MRD检测结果应用于临床的指南或共识。为推动MRD在AML诊疗中的规范化应用、提高诊治水平，中华医学会血液学分会实验诊断学组组织国内AML诊疗领域的相关专家，结合国内外相关研究进展，制定了AML患者MRD检测与临床解读的中国专家共识。该共识包括：MRD的概念和检测方法、MFC和分子生物学方法检测MRD、检测结果的临床解读等部分，具体如下。

一、MRD的概念和检测方法

就AML而言，MRD是指初诊或难治/复发状态的患者经化疗、靶向治疗、嵌合抗原受体T细胞和（或）异基因造血干细胞移植（allo-HSCT）等治疗获得血液学完全缓解（CHR，骨髓涂片经瑞特-吉姆萨染色，光学显微镜检测原始细胞<5％）后体内残存的少量白血病细胞[19]–[21]。健康成人骨髓有核细胞数为（10～13）×10^9^/kg；一个体重70 kg的患者骨髓有核细胞数量为（0.7～0.9）×10^12^，如果以骨髓细胞形态学的5％为阈值，那么AML患者获得CHR时，体内还剩余10^10^左右的白血病细胞[19]–[20]；与CHR相比，MRD检测的敏感性为10^−2^～10^−6^（图1）。AML患者获得CHR并继续接受治疗后的转归如下：①极少数患者发生早期复发；②部分患者呈MRD持续阳性状态，最终发生晚期复发；③部分患者由MRD阳性进入MRD阴性状态，即用现有的MFC、RQ-PCR和NGS等方法在患者体内检测不到白血病细胞；部分MRD阴性患者可再次转为MRD阳性导致晚期复发；部分患者获得临床治愈（图1）。现今，国内外学者尚不清楚AML患者经过诱导治疗、巩固治疗后体内的白血病负荷低于哪个“阈值”就可停止治疗，最终达到治愈的目标。

关于MRD有两个英文名词，即minimal residual disease（微小残留病）和measurable residual disease（可检测到的残留病），前者和上面所述的概念一致[10],[22]；后者是指AML患者经过治疗后，无论获得CHR与否，体内可检测到的白血病细胞。迄今为止，在已经发表的国内外文献中，多数作者通过确定阈值将MRD分为阳性和阴性两种状态[5],[8]–[9],[11]–[15]；然而，由于敏感性所限，文献中所描述的MRD阴性是指用现有方法检测不到患者体内存在白血病细胞，即MRD阴性可以是体内存在白血病细胞但检测不到，也可以是体内已不存在白血病细胞。因此，部分学者提出将MRD阳性和MRD阴性分别称为“detectable MRD（可检测到MRD）”和“undetectable MRD（未检测到MRD）”（图1）[23]。目前，临床常规应用和正在临床研究阶段的AML MRD检测方法见表1。

**图1:**
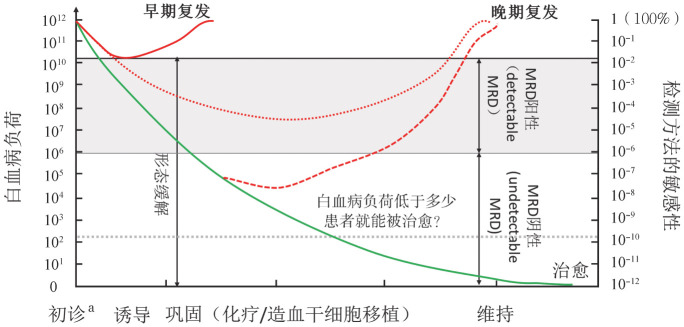
AML患者治疗后MRD检测与疗效/预后模式图 AML：急性髓系白血病；MRD：微小残留病；detectable MRD：可检测到的MRD；undetectable MRD：不能检测到的MRD。^a^除初诊患者以外，难治/复发AML治疗后也需进行MRD评估

（一）MFC检测MRD

临床实践中，用于MFC评估表型异常白血病细胞的单克隆免疫荧光抗体包括CD34、CD117、CD13和CD33，跨系表达抗原CD2、CD7、CD19和CD56等[6]–[7],[9]–[11],[13],[15]；需要注意的是，以CD7等标志进行MRD检测和分析时应考虑其在正常造血细胞上的表达情况。利用MFC检测MRD的方法包括白血病相关异常表型（LAIP）以及与正常骨髓细胞表型相鉴别（D-F-N，different from normal），前者是指初诊时确定患者的LAIP，在随后的治疗过程中利用LAIP进行MRD检测；后者可用于缺乏初诊LAIP的患者，也可检测治疗过程中出现的抗原漂移（Antigen shift）。LAIP和D-F-N相结合既适用于缺乏初诊LAIP的患者，又可检测新出现的表型异常或LAIP发生的抗原漂移[10],[16],[24]–[29]。本专家共识推荐：联合应用LAIP与D-F-N两种方法检测已知和（或）验证未知的、具有预后意义的白血病细胞表型异常。

**表1:** 不同方法检测AML患者MRD的比较

检测方法	敏感性	常规应用	评价
MFC	10^−3^～10^−5^	是	优点：简便快捷、费用低，适用90％以上的AML人群 缺点：抗原漂移；不能确定特定白血病亚群；分析过程复杂
RQ-PCR			
融合基因/NPM1突变	10^−4^～10^−6^	是	优点：特异性好，敏感性高，相对便宜，易标准化 缺点：耗时间、要求靶基因在疾病治疗过程中稳定表达、适用部分、RNA不稳定；仅适用30％～60％的人群
WT1	10^−2^	是	优点：适用80％～90％的AML人群 缺点：敏感性低、缺乏疾病特异性
dd-PCR	10^−4^～10^−6^	否	优点：绝对定量，不需要标准化曲线 缺点：同RQ-PCR
二代测序技术	10^−3^～10^−6^	否	优点：可以同时检测多种突变位点，可观察克隆演变，高通量 缺点：费用高、结果易受背景噪音影响、无标准化

注：AML：急性髓系白血病；MRD：微小残留病；MFC：多参数流式细胞术；RQ-PCR：实时定量聚合酶链反应；dd-PCR：数字聚合酶链反应

1. 检测技术要求：

（1）流式细胞仪：诊断学组专家推荐应用至少八色荧光标记的MFC对标本进行MRD检测以提高特异性。不同实验室或同一实验室不同仪器应遵循标准化操作流程，参见《多参数流式细胞术检测急性白血病及浆细胞肿瘤微小残留病中国专家共识（2017年版）》[30]。

（2）抗体组合和荧光标记：用于MRD检测抗体组合[6]–[7],[9]–[11],[13],[15],[30]–[31]包括：①骨干抗体有CD45、CD117、CD34、CD13以及CD33；②其他抗体：例如借助CD4、CD11b、CD14、CD64和HLA-DR等评估单核、粒-单核AML的MRD，利用CD7、CD19、CD56等评估跨系抗原表达，应用CD133、CD38和CD123等检测白血病干/祖细胞。

专家组推荐的八色MFC抗体组合如下：CD38-FITC/CD33-PE-Cy5.5/CD34-PE-Cy7/CD13-APC/HLA-DR-APC-H7（或APC-Cy7或APC-AlexaFlour750）/CD117-Bv421/CD45-Pacific Orange，PE通道可选择的抗体包括CD56、CD19、CD2、CD4、CD5、CD7、CD11b、CD64、CD15、CD123、NG2（anti-7.1）、CD10等；对于配备十色MFC的中心，可针对PE、Bv605/ECD或APC-R700三个检测通道从可选择抗体中调配MRD检测十色抗体组合[10],[30]–[31]；各中心应根据AML亚型，可选择1管或多管抗体组合进行MRD检测。专家组建议运用典型LAIP与D-F-N方法检测所有异常表达（包括诊断时和治疗后新增的异常表达），在诊断和随访中应用完整的抗体组合方案。

（3）标本采集储存：①MFC检测MRD所需标本可采用乙二胺四乙酸或肝素抗凝，二者无显著区别。②利用外周血（PB）标本比骨髓（BM）标本检测到的MRD低1个数量级，因此，专家组不推荐应用MFC检测PB标本中的MRD。③为了确保MFC检测的敏感性，避免PB稀释BM标本，专家组建议使用抽取的第一管骨髓液（3～5 ml）进行MRD评估，因为标本中PB含量随BM抽取量增加而增加，易导致BM有核细胞被稀释。如果BM标本中成熟中性粒细胞大于90％，则提示所采集的标本被PB稀释。针对不同的AML患者和治疗的不同时间点，骨髓穿刺操作者采集的BM标本体积应该保持一致。④BM标本运输条件：临床常规检测和（或）多中心协作检测时，在室温条件下BM样本应于3 d内完成抗体标记和检测[10],[30]。

（4）样本抗体标记与检测：MRD检测前的标本准备工作包括：①先行荧光抗体标记，再裂解红细胞，清洗过程中尽最大可能减少细胞丢失；②先裂解红细胞，清洗后再行荧光抗体标记，优点是所有标记的流式管采用相同的操作流程。专家组推荐避光孵育条件下进行抗体标记。

MFC检测MRD的方法参见《多参数流式细胞术检测急性白血病及浆细胞肿瘤微小残留病中国专家共识（2017年版）》的“MRD检测质控和标准化”部分[30]。

2. MRD的识别和评估：关于MFC检测的MRD识别和评估问题，专家组建议使用商用程序，同时确保仪器、设备、抗体组合等的标准化[10],[26]–[28],[30]，本共识具体推荐如下：①如初诊时LAIP明确，则用LAIP测定MRD；如初诊时LAIP不明确，则采用D-F-N方法确定新的LAIP；注意患者之间LAIP的个体化差异。②计算LAIP和（或）D-F-N方法检测的MRD细胞占CD45^+^细胞的比例。③获取有核细胞数量50万～100万（除外CD45^−^细胞和碎片），除在标本获取过程中看到明确的MRD细胞群以外，其他残留白血病细胞亚群需由技术熟练的操作者/具备流式细胞术专业知识的人员来确定。

3. MFC检测的MRD报告方式：MFC检测的MRD出具报告时应注意以下事项[10],[30]–[31]：① LAIP细胞的免疫表型特征及其占BM或PB有核细胞的比例，有条件的单位可报告有核细胞和LAIP的绝对数量。②对于初诊时的LAIP，应计算其占初诊白血病原始细胞的百分比。③临床医师和实验室应协商是否需要确定MRD的阈值，如果没有利用MFC检测到确定的MRD细胞，应该在报告上写明MRD检测阴性。④报告还应包括细胞活性、BM标本的增生程度、是否被PB稀释；如果可检测的细胞数量少，则在报告LAIP时给出检测到的总有核细胞数量。此外，欧洲白血病网（ELN）MRD工作组的共识推荐[10]：如果MFC检测到的MRD<0.1％，应该在报告中写明“利用MFC可定量检测到MRD，但其临床意义不明”。然而，国内多数学者的研究表明MRD阳性（即MRD>0）和阴性（即MRD为0）二分类划分即可很好地预测预后[5],[8]–[9],[11]–[15]。因此，本共识建议在报告中清晰描述MFC检测到的MRD水平即可。

4. MFC检测的MRD临床解读：

（1）MRD阈值的确定：ELN MRD共识推荐MFC检测AML患者MRD的阈值为0.1％[10]。然而，由于阈值受到治疗方法、检测时间点以及标本类型等多种因素的影响，MRD的最佳阈值确定尚存争议。本共识建议国内学者积极开展前瞻性、多中心研究确定不同治疗场景下MFC检测到的MRD阈值，以实现阈值个体化。

（2）临床预后和指导治疗：①诱导和巩固治疗后MFC检测MRD阳性提示复发率高、预后不良；对于无NPM1突变的成年AML患者，第2个疗程诱导治疗后MFC检测MRD阳性的患者可考虑选择allo-HSCT以改善预后[15]。②allo-HSCT前MFC检测到的MRD阳性也提示复发率高、预后不良；我国学者的研究结果提示对于移植前MFC检测MRD阳性的AML，单倍型相合移植的疗效优于HLA相合同胞供者移植[5],[13]。③移植后MRD不仅预测复发，而且指导的抢先干预（例如供者淋巴细胞输注）可以降低血液学复发率，改善移植预后[32]。

此外，专家组的推荐还包括：①不建议缺乏经验的中心开展MFC检测MRD项目；②未来AML患者MFC检测MRD的自动化分析方法很重要，值得研究；③白血病干细胞（LSC）是AML复发的根源[33]，应开展临床试验评估LSC在AML患者MRD检测中的价值。

（二）分子学方法检测MRD

MRD的分子学检测方法有PCR和测序技术：PCR包括定性PCR、RQ-PCR、dd-PCR等；测序技术包括Sanger测序和NGS等。虽然RQ-PCR具有较高敏感性，但仅有不足40％的AML患者携带白血病特异性分子标志[16]–[18],[34]–[38]。随着生物信息学等技术的进步，我们极有可能利用NGS等方法解决其他60％左右AML患者的MRD检测问题。专家组推荐RQ-PCR常规用于AML患者的MRD评估；dd-PCR和NGS是否常规用于AML患者MRD的评估尚待今后的研究验证。

1. MRD评估的分子标志：

（1）确定的分子标志：白血病融合基因RUNX1-RUNX1T1、CBFβ-MYH11和PML-RARα以及NPM1突变在治疗后的持续存在是预测AML复发的可靠分子标志，RQ-PCR检测上述标志的敏感性为10^−4^～10^-6^[4],[12],[14],[34]–[38]。获得CHR的AML患者体内可检测到DNMT3A、ASXL1和TET2等前白血病克隆相关突变，由于这些突变也可见于健康人群，且发生率随年龄增大而增加，也被称为年龄相关的克隆造血或意义未名的克隆性造血（CHIP）[39]–[41]，因此，这些突变的复发预测意义仍待证实。在AML患者中，上述突变常发生在恶性转化的极早期阶段，对一些晚期发生的获得性突变，目前还不能确定其能否作为AML MRD检测的可靠标志。

对于更多的AML患者来说，泛白血病基因——WT1可作为缺乏特异性基因的AML患者的MRD标志[7]。在中国，尚缺乏条件对每例初治患者进行全基因组测序来找到个体特异性分子标志，因此，WT1成为实用、快捷的MRD监测指标。初诊时，80％～90％的AML患者伴WT1表达升高；对缺乏特异性基因标志的患者而言，可在诱导缓解后、巩固治疗和结束治疗后以及移植前后单独利用WT1，或联合MFC评估MRD水平[42]。

（2）其他分子标志：由于FLT3-ITD、FLT3-TKD、NRAS、KRAS、IDH1和IDH2等部分基因突变在AML患者复发时存在不稳定现象（如初诊阳性突变，复发时可阴性，或初诊阴性而复发时新出现），这些分子标志不适合作为单独的MRD标志；但上述分子异常与其他MRD标志物联合应用时，可明显降低假阳性和假阴性率[10],[16]–[18],[34]–[38]。此外，包括RUNX1、GATA2、CEBPA、DDX41和ANKRD26在内的某些胚系基因突变与AML发生风险相关[10]，如果把这些基因突变作为MRD评估的标志，就需要应用DNA测序的方法除外它们是胚系组织（皮肤组织、毛囊或颊黏膜）来源的突变[43]。如果治疗后原始细胞数量下降，但基因突变的等位基因变异频率（VAF）并未随肿瘤负荷平行下降，应根据基因的种类考虑胚系基因突变或CHIP的可能性。

多个MRD分子标志物组合应用可克服单个分子标志物评估MRD的缺陷，该缺陷包括AML亚克隆的异质性和CHIP的存在对MRD的影响等[10],[44]–[45]。NGS检测MRD的进展使借助组合标记检测MRD变得切实可行[16]–[18]。例如，AML患者可存在TP53、ASXL1和PTPN11突变；在患者获得CHR状态后，高VAF的ASXL1突变可能由克隆造血所致，不能被用来评估MRD；PTPN11突变克隆可被化疗成功清除；此时，TP53克隆的持续存在可能是复发的根源[10]。因此，同时分析数个分子标志有助于提早预测分子复发。在接受allo-HSCT的患者中，受者造血系统肿瘤相关的胚系突变和CHIP相关突变未来也可能成为移植后MRD评估的标志[40]。

2. 分子标志检测MRD的技术要求：专家组推荐cDNA而非DNA用于基因检测；RUNX1-RUNX1T1、CBFβ-MYH11和PML-RARα融合基因的具体检测方法参见文献[34]–[35]。根据欧洲抗癌计划标准[34]–[35]，每个样本应同时进行3个反应，PCR结果阳性定义为3个重复反应中至少2个反应Ct值≤40［循环阈值（CT）为0.1］。作为对照，专家组建议同时包括野生型样品（正常对照），以及至少2个覆盖所需灵敏度范围的阳性对照和非目标对照（水对照）。如果阳性对照是从质粒产生的，则定期监测质粒稳定性。

PCR方法检测MRD时，推荐应用1 µg RNA逆转录为cDNA，每次反应的cDNA相当于100 ng RNA（约为200 000个细胞，如果基因表达水平高则需要的细胞数量少）。对于基于DNA的方法，每次反应需要至少100 ng DNA（约为15 000个细胞），理想目标是每次反应达到1000 ng（约为150 000个细胞）。管家基因ABL的拷贝数至少应该为10 000拷贝（无论是基于RNA还是DNA的方法）。1000～9999 ABL拷贝数的管家基因数量也可以报告MRD结果，但需要特别标明管家基因拷贝数低于理想值[34]–[35]。

MRD从阴性转为阳性后，应使用2种方法来控制重复样品中的检测变异性：其一，在复核检测的样本中，应包括可疑分子复发的初始样本；其二，如果MRD检测采用RQ-PCR方法，标准曲线应覆盖到患者样本可能的CT值范围，以确保检测的MRD水平在此线性范围内。如果MRD结果为阴性，确认检测的敏感性至关重要[34]–[35]。在计算单独一次RQ-PCR结果的敏感性时建议应用下面的公式，它可用于绝对定量，即利用外源性质粒标准品估计目标分子的个数，和相对定量一样。

X＝［（CT_目标基因_−CT_ABL_）_FU_−（CT_目标基因_−CT_ABL_）_诊断_］/斜率

方法的敏感性＝10^X^

公式中ABL指管家基因ABL；诊断指诊断时进行MRD检测；FU指随访时进行MRD检测；斜率指标准曲线的斜率，如果方法的效率为100％，则斜率为−3.32；目标基因指MRD检测的目标基因。

3. 分子学MRD结果报告方式：各临床中心血液诊断实验室出具分子学MRD报告时应注意以下事项[10]：①写明MRD检测的靶基因，如NPM1、CBFβ-MYH11等；②标明所应用的技术，如RQ-PCR；③标明标本来源，如BM或PB等；④标本质量是否适合行MRD检测；⑤靶基因的定量，如CBFβ-MYH11基因的拷贝数和CT值；⑥对照基因，如ABL和其CT值以及定量；⑦标明检测方法的敏感性等。此外，有条件的单位可以给出所检测基因预测AML患者治疗后复发的阈值，并就下次复查的时间给出建议。

4. 分子学MRD的临床解读：

（1）分子学MRD的概念[10]：①完全分子学缓解（CR_MMRD−_）：定义CR_MMRD−_的前提是患者必须获得CHR，连续两次分子学MRD阴性，标本采集间隔时间≥4周，检测方法敏感性至少为10^−3^。②低水平分子标志持续存在：指患者处于CHR，分子生物学MRD标志持续低水平存在和治疗结束后任何两份阳性标本之间基因检测的拷贝数相对上升<1个数量级。③分子学进展：低水平分子标志持续存在患者，任何两份阳性标本之间MRD标志基因检测拷贝数升高≥1个数量级。④分子学复发：患者处于CHR且CR_MMRD−_后，再次出现MRD阳性，两份阳性标本之间MRD水平上升≥1个数量级。

（2）临床解读：

①急性早幼粒细胞白血病（APL）：对于APL患者而言，最重要的MRD终点是巩固治疗后RQ-PCR检测PML-RARα融合基因为阴性；治疗过程中PML-RARα阳性不是更换治疗方案的指征；治疗结束后，PML-RARα从检测不到至可检测到（PB/BM作为标本，重复两次检测阳性）预警APL血液学复发[37],[46]–[48]。

对于Sanz积分为低、中危，并接受全反式维甲酸（ATRA）联合蒽环类药物治疗的患者，临床医师应在诱导治疗后检测BM MRD；在缓解后的2年内每3个月检测1次BM或PB中的MRD。如果患者接受ATRA+砷剂治疗，临床医师就要持续检测BM MRD直到患者获得CR_MMRD−_，然后停止检测。对于Sanz积分高危患者而言，临床医师应在治疗期间每3个月检测1次PB或BM的MRD，持续至少2年[46]–[49]。大约1/3的APL患者可检出FLT3突变，但不影响APL治疗，也无需对该突变进行持续检测。

②CBFβ-MYH11阳性AML：该亚型AML患者2个疗程巩固治疗后和化疗结束后CBFβ-MYH11检测水平与疾病复发相关。对于巩固治疗2个疗程后CBFβ-MYH11/ABL水平>0.1％的AML患者而言，allo-HSCT可使他们获益[50]。值得注意的是该亚型患者巩固治疗过程中可能持续检测到CBFβ-MYH11转录本的低水平、稳定存在，但并不一定提示疾病复发[51]。

③RUNX1-RUNX1T1阳性AML：对于伴t（8;21）的 AML患者而言，巩固治疗2个疗程后RUNX1-RUNX1T1转录本下降>3个数量级是预后良好的标志，复发率明显低于下降≤3个数量级的患者[12],[52]。由于RUNX1-RUNX1T1转录本由阴性转为阳性至血液学复发时间较短，建议治疗过程中及结束治疗后早期每月检测1次。北京大学血液病研究所的前瞻性临床研究结果显示对于巩固治疗2个疗程后转录本下降<3个数量级的患者，后续接受allo-HSCT可以改善预后[12]。

④伴有NPM1突变的AML：诱导治疗2个疗程后PB标本中NPM1突变的存在提示复发率高，预后不良[14],[38]。如果治疗结束后PB NPM1突变阴性，但BM阳性，专家组建议每4周检测PB/BM NPM1突变1次，至少持续3个月。如果NPM1突变水平上升1个数量级，就应该启动挽救治疗。如果NPM1突变水平检测不到，则应该在治疗结束后的2年内，每3个月评估1次MRD[45],[53]。

⑤其他分子标志：关于其他分子标志物（如BCR-ABL1、WT1、FLT3-ITD、RUNX1、KMT2A-MLLT3或 KMT2A-ELL等）的阈值和检测的时间点还没有形成共识[54]–[55]。

二、AML患者MRD检测的其他推荐

（一）推荐临床试验中的MRD检测

无论是化疗还是allo-HSCT场景下，获得CHR后MRD阳性的AML患者都是复发高危人群，这些患者应该考虑进入临床试验[56]–[58]，并以明确现有方法或试验性方法能否改善MRD阳性患者的预后作为临床试验的目标。专家组建议对于纳入临床试验的患者，应遵循本共识的推荐方法在疗效评估的每个时间点进行MRD检测。

（二）以MRD作为替代终点加速药物获批

MRD在临床实践中预警复发、指导治疗方案选择的价值已经很明确，但是在AML临床试验中将MRD作为替代研究终点还需更多的证据[59]–[60]。如果MRD阴性作为生存的替代终点，对新药评价极有帮助，可能加速有效药物获批，提前终止疗效不佳药物的临床试验。目前2项研究强烈提示MRD可以作为生存的替代终点，在核心结合因子相关AML（CBF-AML）临床研究中，大剂量柔红霉素改善临床预后与MRD水平密切相关[59]；另一项研究中，初诊AML患者的诱导方案中加入维奈克拉改善预后也与MRD状态密切相关[60]。

三、MRD检测的标本和时间点

在AML治疗期间，MFC以及分子生物学MRD评估的时间点包括初诊、2个疗程标准诱导治疗后、巩固治疗后和治疗结束后[6]–[7],[9]–[11],[13],[15],[30]–[31]。对于接受allo-HSCT的患者而言，应在末次化疗结束后（或在预处理前的4周内）采集BM和（或）PB行MRD检测。建议有条件的中心可在患者诱导、巩固治疗的每个疗程结束后评估1次MRD；移植后半年内每个月评估1次MRD，移植后半年到2年内每3～6个月评估1次。此外，对于结合临床表现怀疑患者疾病的任意时间点均推荐进行MRD的检测。

推荐应用BM标本进行MFC检测MRD；推荐应用BM（首选）或PB（次选）标本进行分子学MRD检测。

四、小结

总之，在过去的十年余中，MRD检测在AML患者疗效评估、复发预警以及指导干预和治疗方法选择等方面取得很大进展，但白血病复发仍然是AML患者治疗后最主要的死亡原因之一。规范MRD检测、准确解读MRD的临床意义并指导个体化治疗对降低AML治疗后的复发率、改善生存至关重要。随着今后在AML诊疗领域MRD研究和临床实践的进展，本共识将不断更新、逐步完善。
